# User factors affecting the use of digital services in five European regions and countries

**DOI:** 10.1038/s41597-024-03318-9

**Published:** 2024-05-08

**Authors:** Joy Goodman-Deane, Sam Waller, Mike Bradley, P. John Clarkson, Boris Lazzarini, Elisabet Roca Bosch, Silvia Gaggi

**Affiliations:** 1 Engineering Design Centre, Department of Engineering, University of Cambridge, Cambridge, CB2 1PZ UK; 2 Institute for Sustainability Science and Technology, Universitat Politècnia de Catalunya, Barcelona, Spain; 3 Isinnova – Institute of Studies for the Integration of Systems, Via Sistina 42, 00187 Roma, Italy

**Keywords:** Information technology, Technology

## Abstract

Digitalisation has great potential to reduce costs, improve access and enhance user experience. However, it could also increase inequality, with some people struggling to access and use digital services. It is important to understand who is likely to be excluded in this way and why. This can help to identify groups at particular risk of digital exclusion, inform efforts to overcome the barriers, and develop more inclusive digital services. This paper introduces a set of five linked datasets examining a range of user factors affecting the use of digital services. The datasets focus on the use of digital mobility services, but the data is useful in understanding the use of other digital services as well. The user factors considered include technology access, use and competence and attitudes towards digital technology. The datasets were the results of surveys in five European regions and countries (Germany, Italy, Barcelona Metropolitan Area, Flanders and the Netherlands). Samples were taken of the adult (age 16+) population with a total of 3,454 participants.

## Background & Summary

Technological advances, such as digitalization, smartphones, augmented reality and smart applications, offer great potential to improve people’s lives and experiences. They can provide better access to information and support, offer new functionalities and provide information and interfaces customised to particular users’ needs. For example, in the realm of public transport, digital offerings can combine multiple transport modes more seamlessly, assist with route planning and provide on-demand public transport, as well as offering new business models^1,2^.

However, many of these services require the end user to use a digital interface in order to access them. Users who cannot do so for one reason or another may end up being excluded from using the service. This is a particular issue for public sector services, such as public transport and healthcare provision, because many of those who could benefit the most from improved access to these services are also at higher risk of digital exclusion. For example, Durand *et al*.^3^ identified age, income, education levels, ethnicity, gender and location as aspects playing a role in digital exclusion from transport services. Groth^4^ further explained how ‘marginalised people lack not only well-known mode options such as a car or season tickets for public transport but also appropriate ICTs as key access media to smart mobility’.

It is therefore important to consider the type and extent of digital exclusion when commissioning and designing digital services. Data on this could be useful to diverse categories of professionals, such as local authorities, policy makers, transport and healthcare providers, and app and web designers.

To this end, this paper presents a set of five linked datasets examining a range of user factors affecting the use of digital services. The user factors include various aspects of digital technology access, use and competence, as well as attitudes towards digital technology. Information on limitations in physical, sensory and cognitive capabilities and demographics was also collected. The data was gathered as part of the DIGNITY project^5^ (see also https://www.dignity-project.eu/), which focused on inclusive digital mobility services. As a result, one module in the questionnaire focused on digital transport, while the rest examined factors that affect the use of digital products and services in general.

The datasets were gathered using in-person questionnaires with samples taken from the adult (age 16+) population in five European regions and countries (Germany, Italy, the Barcelona Metropolitan Area, Flanders and the Netherlands), with a total of 3,454 participants. The survey in each region was conducted by a local market research/survey company, under the direction of a research partner in that country. As a result, there was some variation in the sampling and recruitment strategies in each region (see the ‘Methods’ section of this paper for details).

The majority of the questionnaire was composed of multiple-choice self-report questions. The exceptions were the module on technology competence and the assessment of vision ability. Technology competence on eight basic digital interface tests was measured using a simplified paper prototyping method and vision ability was measured using a vision chart. Further information is provided in the ‘Methods’ section.

The main goal of gathering this data was to improve the understanding of digital exclusion in transport in a variety of countries across Europe. In addition, the data can be useful for understanding digital exclusion in other sectors, such as healthcare, online banking and government services. By examining a range of user factors, it provides a more nuanced and multi-faceted understanding of the different ways in which people could be excluded and thus the measures needed to address this exclusion.

The data can also be used to estimate the proportion of the population that would be excluded from using a particular digital service or product by examining the numbers of people in the surveys who do not have sufficient technology access, skills or attitudes to use it^6,7^. This can be helpful for comparing alternative options for services and identifying ways in which they could be made more inclusive.

Another contribution of the datasets is more accurate data on technology competence. The questionnaires used a paper prototyping method to assess this. This provides a more accurate measure than the methods often used in medium and large-scale surveys, such as self-report questions or estimating competence based on technology use. Although some other large-scale surveys of technology competence have been conducted, notably the OECD’s Survey of Adult Skills^8^, they often survey only the working-age or student population.

The results demonstrate a wide range in technology use, attitudes towards technology and digital interface competence across the population. The large numbers with low levels of these variables highlight the importance of considering digital exclusion when designing products and services for the whole population.

## Methods

### Overall method

A survey was conducted in five different European countries. The surveys in Germany, Italy and the Netherlands examined the whole country. The ones in Spain and Belgium focused on smaller regions within these countries due to significant cultural, language and other differences between regions in these countries and due to the initial intended use of the data. The regions examined were the Barcelona Metropolitan Area in Spain and Flanders in Belgium.

The survey process was co-ordinated and managed by the research partners at the University of Cambridge, UK. The survey in each country was conducted by a local market research/survey company, under the direction of a research partner in that country. As a result, there was some variation in the sampling and recruitment strategies in each country, as detailed in the ‘Sampling’ subsection below.

All of the questionnaires were administered in face-to-face interviews. An online survey was not appropriate because it was important to obtain data from people with all levels of digital experience and competence, including those with no internet connection. Phone interviews were not used because the technology competence questions involved paper mock-ups of smartphone interfaces, as explained in the ‘Questionnaire’ subsection below.

The surveys were conducted in 2020 and 2021. As such, they were affected by the COVID-19 pandemic and consequent social distancing and lockdown restrictions. As a result, they were conducted at different dates in different countries, dependent on the local COVID-19 restrictions. The dates for each country are shown in Table 1. Note that the surveys are listed here and throughout this paper in chronological order of survey completion. The survey in the Netherlands was conducted in multiple phases because additional COVID-19 restrictions were imposed in that country part-way through data collection. A third phase was required to obtain additional interviews to better match the quotas in the quota sampling.

**Table 1:** Dates when the survey was conducted in each region/country.

Region/country	Date of survey
Germany	July – Sep 2020
Italy	Nov 2020
Barcelona Metropolitan Area (Spain)	Nov-Dec 2020
Flanders (Belgium)	June-Sep 2021
The Netherlands	First phase: Sep 2020
	Second phase: July-Sep 2021
	Third phase: Nov 2021

The questionnaire took around 20 to 30 minutes to administer. It was kept short to encourage participation from a wide range of participants.

The method for the German survey is described in previous papers^9,10^ and many of the details are the same for the surveys in the other countries. Full details of the method were also published in a project deliverable (not peer-reviewed)^11^. However, the method is described again here for convenience and clarity.

### Ethics

All relevant ethical regulations were followed. Ethical approval for the surveys was obtained from the University of Cambridge Engineering Department ethics committee.

At the start of each interview, the participants first read an information sheet and gave informed consent to taking part and to their anonymous data being included in a publicly available dataset. They could also decline to answer any or all of the questions if they wished.

Each of the local market research/survey companies were based in and allowed to operate in the relevant country. Each local survey adhered to GDPR (data protection) regulations and local laws on the inclusion of individuals aged 16 and 17. In most of the countries, regulations did not require that parental consent be obtained for individuals aged 16 and over. However, if required in a country (and sometimes even if not required), permission was obtained from a parent or guardian for those aged 16 and 17 to participate in the survey and for the data to collected, stored and shared.

### Sampling

Samples were taken of the adult population (age 16+) in each country or region. Initially the survey was intended to be conducted with 300 to 500 participants in each country, using quota sampling. However, in some countries, it was possible to obtain a larger sample. The sampling and recruitment procedures are detailed for each country in Table 2 below.

**Table 2:** Sampling and recruitment in the different regions.

Region/Country	Sample size	Sampling method and incentives
Germany	1,010	The ADM face-to-face area sampling system (https://www.adm-ev.de/): After the selection of sample locations, the private households to be surveyed and target persons within these households were selected at random using a random route procedure. At least four contact attempts were made for each target household or person. No incentives were offered to participants.
Italy	1,002	Adults aged 18 and over were selected on a random basis from the electoral lists of about 140 municipalities located throughout Italy. Young people aged 15–17 (who were under voting age and thus not on the electoral lists), were selected using quotas of age and gender. Only those aged 16 and over were included in the survey dataset. Interviews were conducted in participants’ homes. No incentives were offered to participants.
Barcelona Metropolitan Area (Spain)	601	Two subsamples were defined: Barcelona city and the rest of the Metropolitan Area. In Barcelona city, the sample was stratified by district. In the rest of the Metropolitan Area, the sample was stratified based on location and the size of the municipality. Within each stratum, municipalities were randomly selected.
		In each district or municipality, individuals were recruited on street. The third person to pass the interviewer from the moment they were free was approached (with the caveat that two people from the same group were never interviewed). Quotas were used to ensure a good representation across the population. In Barcelona city, quotas were set on age, gender and nationality (Spanish or other). In the rest of the Metropolitan Area, quotes were set on age, gender, nationality and type of location (level of urbanisation). The interviews were conducted on street. No incentives were offered to participants.
Flanders (Belgium)	418	Quota sampling was used with quotas on age, gender, location (urban or rural), technology use (smartphone use) and education. Participants were recruited from interviewers’ networks following these quotas. Interviews were conducted in participants’ homes and an incentive was offered in the form of a pen in a special case.
The Netherlands	423	Participants were recruited on street at a railway station and in shopping malls with quotas set on age, gender, education and technology use. Interviews took place at a mobile test centre. Participants were offered an incentive of a 10 Euro gift card for taking part.

### Questionnaire

The survey questionnaire was adapted from a previous survey conducted in the UK in 2019^12^. Some questions were omitted or modified based on the experiences in the UK survey and subsequent validation test^13^. A module was added focusing on the use of technology for transport.

The questions covered a range of topics as described below. Most of the questions were multiple-choice self-report except for Module D (Digital interface competence).

- **Module A: Technology access and use**. Participants were asked multiple-choice questions about their access to and frequency of use of the internet, computers, tablet devices and smartphones. Questions were based on items in the Office for National Statistics Internet Access Survey 2017^14^ to allow for comparison with UK national statistics.
- **Module B1: Technology for transport**. Participants rated their confidence in their ability to plan an unfamiliar, local public transport journey using a computer and then using a smartphone, on a scale from 1 (Not at all confident) to 10 (Totally confident). Multiple-choice questions then examined what sources (both digital and non-digital) participants used to obtain information about public transport and how often participants used specific digital transport services (car sharing, car pooling, digital taxi services, on-street bike hire, on-street scooter/motorbike hire and mobile phone parking payment). Participants were also asked whether they felt limited in their regular travel within their region (mobility poverty) for any of the following reasons: cost of travel, limited availability of transport services, limited availability of infrastructure, concerns about safety, difficulty due to special needs/disabilities, digital skills being needed to plan travel, digital skills being needed to use the transport and any other reasons.
- **Module B2: General computer and mobile device activities**. Participants were asked whether they had performed various technology activities recently, using questions based on ones in the Internet Access Survey 2017^14^. The specific activities were adjusted to be more applicable to digital transport services. In order to match the Internet Access Survey 2017, interviewees were first asked if they had performed a set of activities for personal use in the previous 3 months and were then asked about a second set of activities for either personal or work use in the previous 12 months. The first set (activities in the previous 3 months) were e-mail, video/voice internet calls, social networking, online news, internet search, finding information about goods/services, buying goods/services, internet banking, booking travel, using mapping applications and other travel services. The second set of activities were moving/copying files, moving a file between devices, installing software or applications on a computer, installing an app on a tablet or smart[phone, changing software settings, word-processing, editing photos, videos or audio files and writing code.
- **Module C: Attitudes towards technology**. This module was split into two to avoid participant fatigue due to the similarity of the question format. The first set of questions (Module C1) was administered after Module B2, followed by Module D and then Module C2. Module C1 examined overall attitudes towards technology using the ATI (Affinity for Technology Interaction) scale which examines ‘whether users tend to actively approach interaction with technical systems or, rather, tend to avoid intensive interaction with new systems’^15^. Module C2 explored attitudes further using additional questions developed by the authors as part of the 2019 UK survey^12^. The questions are shown in Table 3. Questions 1 and 3 examined the participant’s willingness to explore an unfamiliar interface, which is often important for successful use of a novel technological system. All questions in modules C1 and C2 used the same six-point response scale from “completely disagree” to “completely agree”.

**Table 3:** Questions examining further attitudes towards technology.

**Please indicate the degree to which you agree/disagree with the following statements**
1. When I’m not sure what to do next on a technical system, I try out different things until something works.
2. I need to be shown how to use a technical system many times before I’m confident about using it.
3. I am uneasy about tapping or clicking on things that I don’t recognise in case something breaks.
4. If I tap on the screen or press a button and something happens that wasn’t what I expected, I can usually sort it out by myself.
5. If my current technical system works fine for what I want to do, I have no interest in getting a new one.
- **Module D: Digital interface competence**. This assessed participants’ performance on eight basic digital interface tests using a simplified paper prototyping method. More information on these tests is given in the ‘Performance tests’ subsection below.
- **Module E: Capabilities**. Module E examined participants’ sensory, motor and cognitive capabilities, as these can have a large impact on a user’s interaction with an interface. It was necessary to keep this section short as it was not the main focus of the survey. As a result, most of the questions were self-report, examining how limited participants felt in their daily lives because of issues with various capabilities. In addition, comfort near visual acuity was tested directly using a Snellen vision test chart.
- **Module F: Demographics**. Participants answered questions on their age, gender, social grade or income, education, general health, what type of area they live in (urban or rural) and whether they were an immigrant to the country. These topics were chosen to correspond to key groups that had been identified in previous literature as being at higher risk of digital mobility exclusion.

### Performance tests

**Fig. 1:**
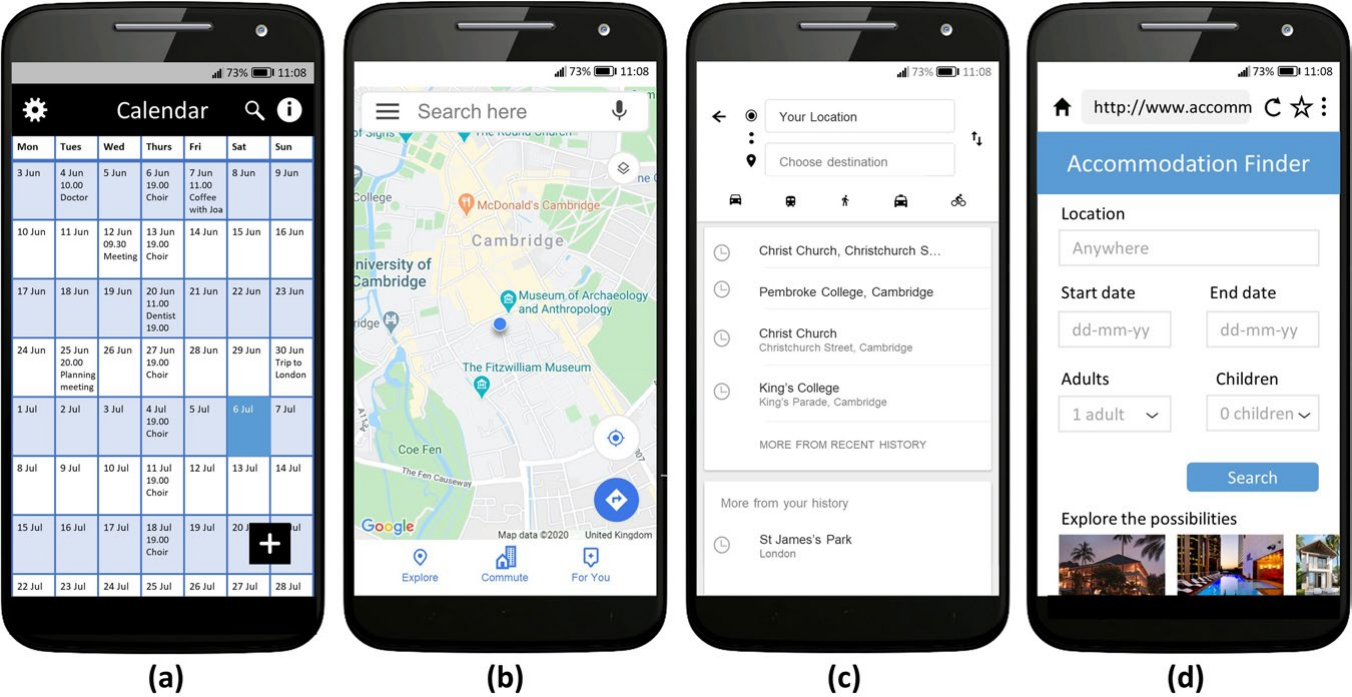
Interfaces used in the digital interface competence tests. Mock-ups of: (**a**) a calendar application, (**b**) a mapping application based on Google Maps, (**c**) a location choice screen within a mapping application, (**d**) a website for finding accommodation.

As mentioned above, Module D assessed participants’ performance on eight basic digital interface tests using a simplified paper prototyping method. In each test, the participants were shown a picture of a smartphone interface on a paper showcard and asked what they would do to achieve a particular goal. The showcards were created in English, based on those in the previous UK survey^12^, and then adapted for use in the different countries with different languages and locations. The English versions of the showcards are shown in Fig. 1.

Participants were asked to indicate on the showcard what they would do to achieve a particular goal. For example, one of the goals for the interface in Fig. 1a was to search for a particular event in the calendar. In some cases, achieving a goal would require several actions. Participants were asked to indicate just the first action they would do. The interviewer coded each response as one of a set of predetermined options such as: Tapped on the magnifying glass symbol, tapped on the settings symbol, scrolled (placed finger on interface and moved it up/down or left/right), something else, said ‘I don’t know’ or preferred not to answer.

This simplified paper prototyping method was used to keep the length and cost of the interviews down, enabling a larger sample size. It was based on the method used by the authors in a previous survey to examine performance on mobile phone menu interfaces^16^.

The set of goals were chosen to cover a range of common, basic interface patterns on a smartphone. They were: search for a particular event in the calendar; change the settings, such as the coolers used in the calendar; create a new event in the calendar on the 6th July; see a menu with more options; get back to the previous screen; change the number of adults in the accommodation search; make an onscreen keyboard appear so that you can enter a location in the search; set this webpage to be one of your bookmarks or favourites so that you can find it easily later on. As such, the tests examined a basic level of digital interface competence, rather than the capability to perform complex tasks on a digital device.

### Translation and changes to the questionnaire in each country

The questionnaire was developed in English and then translated into the local survey languages (German, Italian, Catalan and Dutch/Flemish) by professional translators. These versions were then translated back into English and checked by the survey creators before the translations were finalised. The demographic questions were adjusted for suitability within each country. Some minor adjustments were also made to account for different examples of technology commonly used in each country.

In addition, there were some inadvertent differences in some of the questionnaires as described below.

- In the German questionnaire, Module B1 q3 (about sources of travel information) was specified to refer only to public transport. 20.7% of the sample said that they did not use public transport and therefore did not describe their sources of travel information.
- In the Flanders survey, there was an error in the routing of the questionnaire for Module B2. This meant that many interviewees were not asked the questions in Module B2 when they should have been. Consequently, the results for Module B2 are invalid for this dataset and have been removed from the distributed Flanders dataset.
- There was a delay in distributing the vision test chart to the Dutch survey company due to postal difficulties. This meant that the first batch of interviews was conducted without the vision test. As a result, there was missing data on the vision test in Module F for 22.3% of the weighted sample (22.2% of the unweighted sample).

### Data processing

Weighting variables were calculated for four of the five datasets to better match the population distribution in the regions. The weighting variables for Germany, Italy and Flanders were calculated to take age, gender and region into account. The weighting variable for the Netherlands took age and gender into account. No weighting variable was calculated for the Barcelona dataset because the dataset already had a good match to the quotas.

The collected data was provided by the survey companies in SPSS format. The raw datasets provided in the data repository^17–21^ were produced from this by renaming the variables to be consistent across the datasets from the different countries. In addition, the variable and value labels were tidied up and changed into English.

The raw datasets were processed further to produce the basic datasets in the data repository^17–21^. This was done using SPSS syntax files which are also provided in the repository. These syntax files performed various functions as described below.

The questionnaire used routing so not all the respondents were asked all questions. For example, if a respondent said that they had never used a computer, then they were not asked how often they used one. The syntax files created variables that are consistent across all respondents based on their answers to the routing questions as well as the final questions. In the example above, the frequency of computer use for the respondent would be coded as ‘Never’.

The responses on the digital interface competence tests were categorised into Correct and Incorrect, and a variable was created with the total number correct.

Missing values were set for each variable. All instances of ‘Prefer not to answer’ were coded as missing. Responses of ‘Don’t know’ were coded differently depending on the kind of question. For example, in questions about technology access, ‘Don’t know’ was coded as ‘No’. The rationale for this was that if someone does not know if they have access to a technology, then for practical purposes, they will probably not be able to access it. Similarly, in the performance tests (with questions like ‘What would you do to achieve this goal?’), ‘Don’t know’ was coded as ‘Incorrect’. If someone is too hesitant to guess a response, then it is likely that they would be unable to achieve the goal in practice on a similar smartphone interface. In most other questions (e.g., ‘How often do you use a computer?’), ‘Don’t know’ was considered to be a missing response.

When a variable was derived from multiple source variables, the derived variables were usually considered to be missing if any of the source variables were missing. The exception to this was the total number of performance tests conducted correctly. This was set to missing if there were 4 or fewer valid answers (out of 8) on individual tests. If there were 5 or more valid answers, then it was set to the number correct. The rationale for this was that if the participant did more than half of the tests, then they were unlikely to have a general objection to the module (e.g., on the grounds of time) and it was more likely to be due to issues and difficulties with the individual tests.

After this processing, variables were created marking specific groups that are more likely to experience digital mobility exclusion (e.g., older people, rural inhabitants, people with low income). The (small number of) open text variables were removed from the publicly available versions of the datasets in case they contained any potentially identifying information.

Excel versions of the datasets were generated by exporting the basic version, omitting the redundant and dummy variables (at the end of the SPSS file) and adding weighted frequency counts for each variable.

## Data Records

The datasets and associated files from all the surveys are stored on the UPCommons repository, with one repository entry for each region’s survey^17–21^. The files in each repository entry are described below. The files are listed in the order in which they were used in the survey, with English versions of the survey materials (questionnaire and showcards) first, followed by the translated versions, the raw datasets and their codebooks, the syntax files for processing the raw datasets, and finally the basic processed versions of the datasets. The file names are given in bold. Note that frequency graphs for all of the main variables can be found in a project deliverable^11^.

### Germany data record

Please note that some of the analysed results from the German survey and a short description of the method used in this survey^17^ were previously published in conference papers^9,22,23^ and a journal paper^10^. The current paper expands on these by providing more details on the files in the data repository entry, the method and technical validation, as well as placing it in the context of the other four surveys.

- **ENGLISH questionnaire.pdf**. PDF file. The English language original version of the questionnaire used in the survey.
- **ENGLISH showcards.pdf**. PDF file. The English language original version of the showcards used in the survey.
- **vision test chart with printing instructions.pdf**. PDF file. The vision test chart used in the survey, with printing instructions in English.
- **GERMANY questionnaire.pdf**. PDF file. The German language version of the questionnaire.
- **GERMANY showcards.pdf. PDF file**. The (German language) showcards used in the survey. Note that the maps and locations on showcards G and H were adjusted to use locations local to the survey.
- **GERMANY RAW weighted with new variable names v02 anonymised.sav**. SPSS data file. The raw version of the dataset after the variable names were standardised across the datasets and the variable and value labels were tidied up and translated into English. It contains 1010 rows (i.e., respondents) and 130 columns (i.e., variables).
- **GERMANY RAW v02 anonymised codebook.pdf**. PDF file. Codebook for the RAW version of the dataset.
- **GERMANY combined syntax.sps**. SPSS syntax file. Syntax for creating the BASIC version of the German dataset from the RAW version.
- **GERMANY BASIC weighted processed dataset v08 anonymised.sav**. SPSS data file. The version of the dataset after further basic processing. It contains 1010 rows (i.e., respondents) and 218 columns (i.e., variables).
- **GERMANY BASIC v08 anonymised codebook.pdf**. PDF file. Codebook for the BASIC version of the dataset.
- **GERMANY BASIC anonymized v08.xlsx**. Excel file. The BASIC version of the dataset in Excel format.

### Italy data record

- **ENGLISH questionnaire.pdf**. PDF file. The English language original version of the questionnaire used in the survey^18^.
- **ENGLISH showcards.pdf**. PDF file. The English language original version of the showcards used in the survey.
- **vision test chart with printing instructions.pdf**. PDF file. The vision test chart used in the survey, with printing instructions in English.
- **ITALY questionnaire.pdf**. PDF file. The Italian language version of the questionnaire.
- **ITALY showcards.pdf. PDF file**. The (Italian language) showcards used in the survey. Note that the maps and locations on showcards G and H were adjusted to use locations local to the survey.
- **ITALY RAW weighted with new variable names v02 anonymised.sav**. SPSS data file. The raw version of the dataset after the variable names were standardised across the datasets and the variable and value labels were tidied up and translated into English. It contains 1002 rows (i.e., respondents) and 127 columns (i.e., variables).
- **ITALY RAW v02 anonymised codebook.pdf**. PDF file. Codebook for the RAW version of the dataset.
- **ITALY combined syntax.sps**. SPSS syntax file. Syntax for creating the BASIC version of the Italian dataset from the RAW version.
- **ITALY BASIC weighted processed dataset v07 anonymised.sav**. SPSS data file. The version of the dataset after further basic processing. It contains 1002 rows (i.e., respondents) and 216 columns (i.e., variables).
- **ITALY BASIC v07 anonymised codebook.pdf**. PDF file. Codebook for the BASIC version of the dataset.
- **ITALY BASIC anonymized v07.xlsx**. Excel file. The BASIC version of the dataset in Excel format.

### Barcelona Metropolitan Area data record

- **ENGLISH questionnaire.pdf**. PDF file. The English language original version of the questionnaire used in the survey^19^.
- **ENGLISH showcards.pdf**. PDF file. The English language original version of the showcards used in the survey.
- **vision test chart with printing instructions.pdf**. PDF file. The vision test chart used in the survey, with printing instructions in English.
- **BARCELONA questionnaire.pdf**. PDF file. The Catalan language version of the questionnaire.
- **BARCELONA showcards.pdf. PDF file**. The (Catalan language) showcards used in the survey. Note that the maps and locations on showcards G and H were adjusted to use locations local to the survey.
- **BARCELONA RAW with new variable names v04 anonymised.sav**. SPSS data file. The raw version of the dataset after the variable names were standardised across the datasets and the variable and value labels were tidied up and translated into English. It contains 601 rows (i.e., respondents) and 135 columns (i.e., variables).
- **BARCELONA RAW v04 anonymised codebook.pdf**. PDF file. Codebook for the RAW version of the dataset.
- **BARCELONA combined syntax.sps**. SPSS syntax file. Syntax for creating the BASIC version of the Barcelona dataset from the RAW version.
- **BARCELONA BASIC processed dataset uniformly weighted v10 anonymised.sav**. SPSS data file. The version of the dataset after further basic processing. It contains 601 rows (i.e., respondents) and 222 columns (i.e., variables).
- **BARCELONA BASIC v10 anonymised codebook.pdf**. PDF file. Codebook for the BASIC version of the dataset.
- **BARCELONA BASIC anonymized v10.xlsx**. Excel file. The BASIC version of the dataset in Excel format.

### Flanders data record

- **ENGLISH questionnaire.pdf**. PDF file. The English language original version of the questionnaire used in the survey^20^.
- **ENGLISH showcards.pdf**. PDF file. The English language original version of the showcards used in the survey.
- **vision test chart with printing instructions.pdf**. PDF file. The vision test chart used in the survey, with printing instructions in English.
- **FLANDERS questionnaire.pdf**. PDF file. The Flemish language version of the questionnaire.
- **FLANDERS showcards.pdf. PDF file**. The (Flemish language) showcards used in the survey. Note that the maps and locations on showcards G and H were adjusted to use locations local to the survey.
- **FLANDERS RAW weighted with new variable names v01 anonymised.sav**. SPSS data file. The raw version of the dataset after the variable names were standardised across the datasets and the variable and value labels were tidied up and translated into English. It contains 418 rows (i.e., respondents) and 128 columns (i.e., variables).
- **FLANDERS RAW v01 anonymised codebook.pdf**. PDF file. Codebook for the RAW version of the dataset.
- **FLANDERS combined syntax.sps**. SPSS syntax file. Syntax for creating the BASIC version of the Flanders dataset from the RAW version.
- **FLANDERS BASIC weighted processed dataset v05 anonymised.sav**. SPSS data file. The version of the dataset after further basic processing. Note that the responses to questions in module B2 have been removed as these questions were mis-administered in the Flanders survey. The file contains 418 rows (i.e., respondents) and 217 columns (i.e., variables).
- **FLANDERS BASIC v05 anonymised codebook.pdf**. PDF file. Codebook for the BASIC version of the dataset.
- **FLANDERS BASIC anonymized v05.xlsx**. Excel file. The BASIC version of the dataset in Excel format.

### The Netherlands data record

- **ENGLISH questionnaire.pdf**. PDF file. The English language original version of the questionnaire used in the survey^21^.
- **ENGLISH showcards.pdf**. PDF file. The English language original version of the showcards used in the survey.
- **vision test chart with printing instructions.pdf**. PDF file. The vision test chart used in the survey, with printing instructions in English.
- **NETHERLANDS questionnaire.pdf**. PDF file. The Dutch language version of the questionnaire.
- **NETHERLANDS showcards.pdf**. PDF file. The (Dutch language) showcards used in the survey. Note that the maps and locations on showcards G and H were adjusted to use locations local to the survey.
- **NETHERLANDS RAW weighted with new variable names v03 anonymised.sav**. SPSS data file. The raw version of the dataset after the variable names were standardised across the datasets and the variable and value labels were tidied up and translated into English. It contains 423 rows (i.e., respondents) and 128 columns (i.e., variables).
- **NETHERLANDS RAW v03 anonymised codebook.pdf**. PDF file. Codebook for the RAW version of the dataset.
- **NETHERLANDS combined syntax.sps**. SPSS syntax file. Syntax for creating the BASIC version of the Netherlands dataset from the RAW version.
- **NETHERLANDS BASIC weighted processed dataset v08 anonymised.sav**. SPSS data file. The version of the dataset after further basic processing. The file contains 423 rows (i.e., respondents) and 218 columns (i.e., variables).
- **NETHERLANDS BASIC v08 anonymised codebook.pdf**. PDF file. Codebook for the BASIC version of the dataset.
- **NETHERLANDS BASIC anonymized v08.xlsx**. Excel file. The BASIC version of the dataset in Excel format.

## Technical Validation

### Sampling

The reliability of survey data depends on the survey sampling. This impacts how representative the sample is of the population from which it is drawn. In the surveys presented in this paper, the practical administration of each individual survey was overseen at the local level. As a result, the sampling methods and sample sizes varied between countries as described in Table 2 in the Methods section. Taking each country in turn:

- Germany: This survey used the ADM face-to-face area sampling system described in Table 2. This is a three-stage stratified random sampling framework, which is frequently employed in market, media and social research in Germany (see https://www.gesis.org/fileadmin/upload/SDMwiki/H%C3%A4der_Sampling_in_Practice.pdf). As such it approximates a random sample and produces a fairly robust, representative sample.
- Italy: This survey used random sampling from electoral lists. This also approximates a random sample and produces a fairly robust, representative sample.
- Barcelona Metropolitan Area: This survey used on-street quota sampling. On-street sampling under-samples those who leave the house less frequently. Furthermore, it may be biased by the choice of locations as the selection of people in some locations may be skewed. This was addressed by selecting the locations carefully: the sample was stratified by district and municipalities were randomly selected within the districts. Participants were recruited on street. Furthermore, quotas together with a randomised procedure for selecting participants to approach were used to reduce interviewer bias in participant selection.
- Flanders: This survey used quota sampling from interviewers’ networks. This has the potential for bias because it relies on the interviewers’ and interviewing company’s contacts. Using quotas helped to reduce these biases but it is impossible to set quotas on all relevant variables (e.g., health and disability).
- The Netherlands: This survey used on-street quota sampling at a railway station and in shopping malls. This method under-samples those who leave the house less frequently. Furthermore, the locations used may skew the selection of participants towards those who are more active. Efforts were made to improve the sample by setting quotas on a range of variables (age, gender, education and technology use) comparing these with national statistics.

The variation in sampling between the countries makes cross-country comparison difficult and means that extra caution is needed in interpreting the results from some of the samples. In particular, on-street sampling (used in Barcelona and the Netherlands) under-samples those who leave the house less frequently. This effect was likely to be more pronounced during the COVID-19 pandemic when more vulnerable individuals and those in poorer health were less likely to go out. The choice of locations in the Netherlands (a railway station and shopping centres) may limit the representativeness of its sample further. Sampling through networks (used in Flanders) is also open to bias, as it relies on the interviewers’ and interviewing company’s contacts. As a result, the surveys conducted in Germany and Italy exhibit greater robustness and yield results that are more representative of the population than the Barcelona, Flanders, and Dutch surveys.

Furthermore, regardless of what type of sampling is used, certain types of people are less likely to take part in a survey and may therefore be under-represented. This includes people with cognitive difficulties for whom answering a survey like this would be difficult. People who are not interested in the topic of the survey (in this case, digital exclusion) are also likely to be under-represented. Efforts were made to mitigate this by emphasizing at the start of the information sheet that we wanted ‘to get a wide range of people from across the adult population, including both people who use technology a lot and people who rarely (or never) use it.’ Comparing the results against national statistics also helped. Nevertheless, some bias remains, and this should be considered when interpreting the survey results. For example, it seems likely that the results will underestimate the numbers of people with cognitive difficulties and with negative attitudes towards technology.

It is also important to recognise that the surveys took place during the COVID-19 pandemic. This may have made some people more cautious about taking part in a face-to-face survey, even though COVID-19 restrictions and guidance were followed. This is likely to disproportionately affect those with underlying health conditions and may thus skew the samples away from older people and those with disabilities. Although quotas were set on age, there may be a greater proportion of healthy and active people among the older segments of the survey samples than in the older population as a whole. The survey results may thus underestimate the difficulties that people have with travel and technology, as well as the numbers of people with disabilities.

**Fig. 2:**
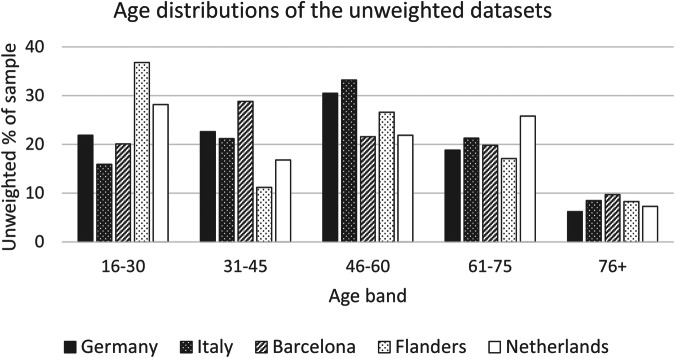
Age distributions of the unweighted datasets.

**Fig. 3:**
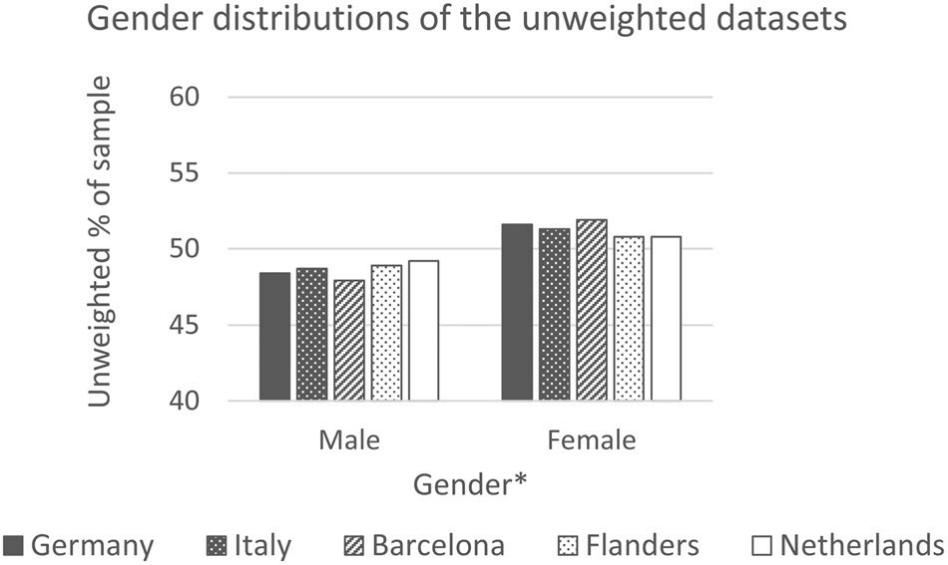
Gender distributions of the unweighted datasets (note the restricted portion of the y-axis shown on the graph). *Note that a small proportion (0–0.2%) of respondents selected the option ‘Prefer to self-describe’ but the proportion is too small to show meaningfully on this graph.

The representativeness of the samples can also be examined by considering their demographic spread. Graphs of the age and gender distributions for the five unweighted survey datasets are shown in Figs. 2, 3. It can be seen that there is considerable variation between the datasets. However, the population age figures available for the different nations/regions did not all use the same age brackets, making it difficult to present a comparison with regional statistics across all the datasets on a single graph. Instead, the comparison is shown for each dataset separately in Tables 4–8.

**Table 4:** Comparison of the German survey sample with national statistics.

Variable	Value	% in German population	% in unweighted sample	% in weighted sample
Age	16–39	33.3%	35.9%	33.4%
	40–64	41.2%	44.4%	41.3%
	65–74	12.0%	12.7%	15.1%
	75+	13.5%	7.0%	10.1%
Gender	Male	49.3%	48.4%	49.0%
	Female	50.7%	51.6%	51.0%
Location	Urban	77.4%	71.0%	70.7%
	Rural	22.6%	29.0%	29.3%
Technology use	Use smartphone at least once a week	81.7%	85.8%	81.9%
	Do not use smartphone at least once a week	18.3%	14.2%	18.1%
Education	Currently attending school	3.5%	1.5%	2.6%
	No school leaving certificate	4.0%	1.7%	2.0%
	School leaving certificate (intermediate or secondary general)	58.6%	71.2%	62.5%
	University entrance qualifications or higher	33.5%	25.7%	33.0%

The population figures for gender and urbanization cover the whole population (age 0 up), those for technology use cover age 14 and over, those for education cover 15 and over.

**Table 5:** Comparison of the Italian survey sample with national statistics.

Variable	Value	% in Italian population	% in unweighted sample	% in weighted sample
Age	18–44	36.5%	33.6%	37.2%
	45–64	36.0%	41.9%	35.6%
	65–74	13.5%	14.7%	17.2%
	75+	14.0%	9.8%	10.0%
Gender	Male	48.7%	48.7%	48.2%
	Female	51.3%	51.3%	51.8%
Technology use	Used the internet in the last 3 months	78%	80.9%	79.5%
	Not used the internet in the last 3 months	22%	19.1%	20.5%
Education	Elementary school or no schooling	16.2%	9.4%	11.3%
	Junior/middle school	32.4%	27.7%	26.2%
	Senior high school or equivalent	36.4%	48.0%	47.4%
	University degree or higher	15.0%	14.9%	15.1%

Location data is not included in this table because it was not possible to ascertain the urban/rural split for this survey as the Italian questionnaire only asked about the size of the commune (region) of residence and many communes include a mix of rural and urban locations.

**Table 6:** Comparison of the Barcelona Metropolitan Area survey sample with regional statistics.

Variable	Value	% in regional population	% in unweighted sample
Age	16–39	34.7%	35.4%
	40–64	42.4%	42.1%
	65–74	11.5%	11.1%
	75+	11.7%	11.3%
Gender	Male	47.5%	47.9%
	Female	52.5%	51.9%
	Prefer to self-describe		0.2%
Technology use	Used a smartphone to access the internet in the last three months	86.5%	84.1%
	Not used a smartphone to access the internet in the last three months	13.5%	15.9%
Education	Primary and lower secondary education (or similar) or lower	45.1%	30.8%
	Higher secondary education	23.2%	15.7%
	Higher education	31.8%	53.5%

A breakdown for urban/rural location is not given for this survey because the area is almost entirely urban. A column for weighted percentages is not included in this table because this dataset is unweighted (see the section of this paper on Data Processing).

**Table 7:** Comparison of the Flanders (Belgium) survey sample with national statistics.

Variable	Value	% in Flanders population	% in unweighted sample	% in weighted sample
Age	16–39	35%	42.0%	31.2%
	40–64	40%	36.1%	43.8%
	65–74	13%	12.0%	13.0%
	75+	12%	10.0%	12.0%
Gender	Male	49%	48.9%	48.9%
	Female	51%	50.8%	50.9%
	Prefer to self-describe		0.2%	0.1%
Location	Urban	45.3%	44.5%	51.0%
	Rural	54.7%	55.5%	49.0%
Technology use	I don’t have a smartphone	7.0%	6.3%	7.7%
	I use my smartphone less than one hour/day	18.0%	18.3%	21.7%
	I use my smartphone more than one hour/day	75.0%	75.5%	70.6%
Education	Primary education and lower secondary education	20.7%	27.2%	24.9%
	Higher secondary education	46.0%	36.3%	36.7%
	Higher non-university and higher university education	33.3%	36.5%	38.4%

**Table 8:** Comparison of the Netherlands survey sample with national statistics.

Variable	Value	% in Dutch population	% in unweighted sample	% in weighted sample
Age	16–39	36.1%	37.5%	35.9%
	40–64	40.2%	37.2%	40.2%
	65–79	18.0%	16.3%	13.9%
	80+	5.8%	9.0%	9.9%
Gender	Male	49.4%	49.2%	49.2%
	Female	50.6%	50.8%	50.8%
Location	Urban	91.9%	82.0%	81.5%
	Rural	8.2%	18.0%	18.5%
Technology use	Used a smartphone to access the internet	88%	91.2%	91.0%
	Not used a smartphone to access the internet	12%	8.8%	9.0%
Education	Primary or prevocational secondary education (LBO, VMBO, MAVO, etc) or below	29%	20.2%	20.0%
	Secondary education (HAVO, VWO, MBO 2–4)	40%	45.9%	46.1%
	Higher or university education (HCO) or above	30%	33.8%	34.0%

Acronyms such as LBO refer to Dutch education levels.

Some of the discrepancy between the sample and the population was addressed by calculating weighting variables for four of the five datasets, as described in the section on ‘Data processing’. Tables 4–8 show the sample distributions for both the unweighted and weighted samples, comparing these to national/regions statistics. They cover age, gender, location (urban/rural), technology use and education level. Technology use is included in the tables because it is particularly important for a survey which is about digital exclusion.

Note that, in some cases, slightly different variables are reported on for different countries, depending on the information available in national statistics for that country.

In the German comparison (Table 4), the population figures for age come from the German Federal Statistical Office via Statista (https://de.statista.com/statistik/daten/studie/1351/umfrage/altersstruktur-der-bevoelkerung-deutschlands/). Those for gender were taken from German census data via Statista (https://de.statista.com/statistik/daten/studie/161868/umfrage/entwicklung-der-gesamtbevoelkerung-nach-geschlecht-seit-1995/). Those for location come from the World Bank, UN DESA via Statista (https://de.statista.com/statistik/daten/studie/662560/umfrage/urbanisierung-in-deutschland/). Population figures for technology use were taken from Vuma Touchpoints (https://www.vuma.de/vuma-praxis/die-studie/ via https://de.statista.com/statistik/daten/studie/585883/umfrage/anteil-der-smartphone-nutzer-in-deutschland/). Population figures for education are for 2019 and were taken from the German Federal Statistical Office: (https://www.destatis.de/EN/Themes/Society-Environment/Education-Research-Culture/Educational-Level/Tables/educational-attainment-population-germany.html).

In the Italian comparison (Table 5), the population figures for age, gender and education are for 2019 and were taken from Statista (https://www.statista.com/). Age figures are given as a proportion of the population/sample aged 18+ because population figures for 16-17 year olds were not available. Population figures for technology use are for 2020 and were taken from Eurostat from the dataset for Digital economy and society (https://ec.europa.eu/eurostat/databrowser/view/isoc_ci_ifp_iu/default/table?lang=en). These figures were available to the nearest whole number. Population figures for education are for 2019 and were taken from Statista (https://www.statista.com/statistics/1088273/population-aged-15-years-and-older-by-educational-level-in-italy/). Figures for vocational qualifications were included in “senior high school or equivalent” to give an estimate for this category. These population figures are for the population aged 15+.

In the Barcelona comparison (Table 6), the population figures for age and gender are for the Barcelona Metropolitan Area in 2020 and are taken from IDESCAT (https://www.idescat.cat). The population percentages for technology use are for Barcelona City and are taken from the 2019 municipal services survey of Barcelona City Council (https://ajuntament.barcelona.cat/ca/informacio-administrativa/registre-enquestes-i-estudis-opinio). To give a good comparison, the survey results for this variable in this table are also reported as a percentage of the participants in Barcelona City (rather than all the participants in the survey). Population percentages for education are those aged 15+ in the whole of Catalonia and are taken from IDESCAT (https://www.idescat.cat). Post-compulsory professional training was included in the ‘Highter education’ category for the survey results. It is unclear whether these were included in the population figures or not.

In the Flanders comparison (Table 7), the population figures were taken from CIM (Centre for Information about the Media).

In the Netherlands comparison (Table 8), the age and gender population data are for 2021 and were taken from CBS (https://www.cbs.nl/nl-nl/visualisaties/dashboard-bevolking/bevolkingspiramide). Population figures for urbanization are for 2019 and were taken from Statista (https://www.statista.com/statistics/276724/urbanization-in-the-netherlands/). Population figures for technology use are for 2019 and were taken from Eurostat from the dataset for Digital economy and society, 2019 (https://ec.europa.eu/eurostat/databrowser/view/isoc_ci_im_i/default/table?lang=en). These figures were available to the nearest whole number. The smartphone use figures from the survey refer to use of a smartphone to access the internet at least once a week. Population figures for education are for 2017 and were taken from https://longreads.cbs.nl/trends18-eng/society/figures/education/. The education levels in the population and the survey were matched as closely as possible, but this may not be exact.

### Questionnaire design

The questionnaire design is described in the ‘Methods’ section. For increased reliability and comparability of the data, several of the survey questions were based on those from previous surveys such as the Office for National Statistics Internet Access Survey 2017^14^. In addition, the attitudes towards technology module (Module C) used the ATI scale which had previously exhibited good to excellent reliability^15^.

The survey questionnaire was adapted from a previous survey conducted in the UK with 338 participants^12^ and followed by a validation test^13^. This means that the majority of questions had already been piloted and tested. Some of the questions were refined after the validation test and before being used in the surveys described in this paper. The questionnaire was also adapted for use in the transport sector. In particular, Module B1 was added. This focuses on the use of technology for transport.

### Other technical validation

All variables that contained conditional routing were examined to make sure that the missing data contained in this variable corroborated with the expected situations where this question was not asked. It was this process that identified that the routing for the Flanders technology activities had been implemented incorrectly, and resulted in the data for this module being removed in the published Flanders dataset.

## Data Availability

The data was processed in SPSS v28. The code (SPSS syntax files) used to generate the processed versions of the datasets from the raw versions are included in the data repository entries for each dataset as described in the Data Records section above. There are no restrictions on access to these files.
